# TGFβ1 Restores Energy Homeostasis of Human Trophoblast Cells Under Hyperglycemia In Vitro by Inducing PPARγ Expression, AMPK Activation, and HIF1α Degradation

**DOI:** 10.3390/cells14010045

**Published:** 2025-01-03

**Authors:** Nihad Khiat, Julie Girouard, Emmanuelle Stella Kana Tsapi, Cathy Vaillancourt, Céline Van Themsche, Carlos Reyes-Moreno

**Affiliations:** 1 Groupe de Recherche en Signalisation Cellulaire (GRSC), Département de Biologie Médicale, Université du Québec à Trois-Rivières, 3351 Boulevard des Forges, Trois-Rivières, QC G8Z 4M3, Canada; nihad.khiat@uqtr.ca (N.K.); julie.girouard@uqtr.ca (J.G.); emmanuelle.stella.kana.tsapi@uqtr.ca (E.S.K.T.); celine.vanthemsche@uqtr.ca (C.V.T.); 2 Centre de Recherche Interuniversitaire en Reproduction et Développement-Réseau Québécois en Reproduction (CIRD-RQR), Université de Montréal, St-Hyacinthe, QC J2S 2M2, Canada; cathy.vaillancourt@inrs.ca; 3 Regroupement Intersectoriel de Recherche en Santé de l’Université du Québec (RISUQ), Université du Québec, Québec, QC G1K 9H7, Canada; 4 Institut National de la Recherche Scientifique (INRS)-Centre Armand Frappier Santé Biotechnologie and Research Centre CIUSSS-Nord-de-l’île-de-Montréal, Laval, QC H7V 1B7, Canada

**Keywords:** adenosine triphosphate, diabetes, extra-villous trophoblast, glucose metabolism, glycolysis, hyper-glucose, oxidative phosphorylation, placenta, transforming growth factor beta 1

## Abstract

Elevated glucose levels at the fetal–maternal interface are associated with placental trophoblast dysfunction and increased incidence of pregnancy complications. Trophoblast cells predominantly utilize glucose as an energy source, metabolizing it through glycolysis in the cytoplasm and oxidative respiration in the mitochondria to produce ATP. The TGFβ1/SMAD2 signaling pathway and the transcription factors PPARγ, HIF1α, and AMPK are key regulators of cell metabolism and are known to play critical roles in extravillous trophoblast cell differentiation and function. While HIF1α promotes glycolysis over mitochondrial respiration, PPARγ and AMPK encourage the opposite. However, the interplay between TGFβ1 and these energy-sensing regulators in trophoblast cell glucose metabolism remains unclear. This study aimed to investigate whether and how TGFβ1 regulates energy metabolism in trophoblast cells exposed to normal and high glucose conditions. The trophoblast JEG-3 cells were incubated in normal (5 mM) and high (25 mM) glucose conditions for 24 h in the absence and the presence of TGFβ1. The protein expression levels of phosphor (p)-SMAD2, GLUT1/3, HIF1α, PPARγ, p-AMPK, and specific OXPHOS protein subunits were determined by western blotting, and ATP and lactate production by bioluminescent assay kits. JEG-3 cells exposed to 25 mM glucose decreased ATP production but did not affect lactate production. These changes led to a reduction in the expression levels of GLUT1/3, mitochondrial respiratory chain proteins, and PPARγ, coinciding with an increase in HIF1α expression. Conversely, TGFβ1 treatment at 25 mM glucose reduced HIF1α expression while enhancing the expression levels of GLUT1/3, PPARγ, p-AMPK, and mitochondrial respiratory chain proteins, thereby rejuvenating ATP production. Our findings reveal that high glucose conditions disrupt cellular glucose metabolism in trophoblast cells by perturbing mitochondrial oxidative respiration and decreasing ATP production. Treatment with TGFβ1 appears to counteract this trend, probably by enhancing both glycolytic and mitochondrial metabolism, suggesting a potential regulatory role of TGFβ1 in placental trophoblast cell glucose metabolism.

## 1. Introduction

Elevated levels of glucose have a significant impact on maternal and fetal interactions as well as on the production of essential hormones that support pregnancy [1]. Studies have shown that high glucose levels are linked to a decrease in the number of active mitochondria and reduced ATP production, which can negatively affect trophoblast function [2,3]. This may result in an increased incidence of pregnancy complications such as preeclampsia, congenital malformations, and miscarriage, posing significant health risks to both pregnant women and fetuses [4].

The placenta is a highly metabolically active organ fulfilling the bioenergetic and biosynthetic needs to support its own rapid growth and that of the fetus [5]. Placental structures consist of specialized epithelial cell types known as trophoblast cells, which are located at the maternal–fetal interface [6,7]. Human trophoblasts arise from the trophectoderm, which, after implantation, differentiates into cytotrophoblast (CT), villous syncytiotrophoblast (VST), and extravillous trophoblast (EVT) cells [6,7]. VST cells form a multinucleated layer on the placental surface and play key roles in hormone secretion, nutrient transport, gas exchange, immunotolerance, and pathogen resistance [8,9]. EVT cells migrate, invade, and embed placental villi into the maternal decidua to anchor the placenta [10,11].

Trophoblasts use glucose as the primary energy substrate. In the absence of appreciable gluconeogenesis, placental glucose transport is the only supply for the fetus [12]. In trophoblasts, glucose is metabolized through a multistep process. The first step, glycolysis, occurs in the cytoplasm and involves the conversion of one glucose molecule to two pyruvate molecules, resulting in a net gain of two ATP molecules [1]. Under anaerobic conditions, pyruvate can be reversibly converted to lactate by the enzyme lactate dehydrogenase (LDH). Pyruvate is typically transported into the mitochondria for further degradation in the TCA cycle, where it is oxidized to acetyl-CoA [5]. This process occurs iteratively, generating one ATP molecule for each acetyl-CoA molecule produced, while also generating reduced nicotinamide adenine dinucleotide (NADH+H^+^) and flavin adenine dinucleotide (FADH_2_). These reduced cofactors act as electron carriers to generate a proton gradient that drives ATP production through oxidative phosphorylation (OXPHOS) in the electron transport chain [5].

Cellular glucose metabolism is regulated by complex signaling pathways that involve various biological factors. Transforming growth factor beta 1 (TGFβ1), a multifunctional cytokine, is one of these factors [13]. It operates through the SMAD2/SMAD3 pathway to regulate the expression of genes involved in different cellular functions. According to previous research, TGFβ1 regulates the activity of essential enzymes and transporters, such as GLUT1 and the hexokinase HK2, which play crucial roles in glucose uptake and glycolysis in different cell types [13]. However, TGFβ1 is also known to induce metabolic reprogramming by promoting mitochondrial ATP production in stromal cells [14]. TGFβ1 expression is detected at the human maternal–fetal interface during early pregnancy, playing crucial roles in regulating immune cell function and maintaining immune homeostasis [15]. Moreover, TGFβ1 plays significant roles in the regulation of trophoblast function, particularly trophoblast invasion and differentiation [15].

Transcription factors, such as hypoxia-inducible factor (HIF), AMP-activated protein kinase (AMPK), and peroxisome proliferator-activated receptor gamma (PPARγ), are considered energy sensors that play significant roles in regulating cellular metabolism [16,17,18]. HIFs are transcriptional heterodimer complexes, consisting of an inducible α subunit (HIF1α, HIF2α, and HIF3α) and constitutively expressed β subunits [16]. Under hypoxic conditions, the α/ꞵ heterodimer binds to the hypoxia response elements (HREs) of the target genes. Under normal oxygen levels, proline residues in HIF1α and HIF2α are hydroxylated by prolyl hydroxylase domain (PHD) proteins, allowing their recognition by a ubiquitin ligase leading to proteasomal degradation [17]. Studies have demonstrated that HIF1α is significantly expressed in the low-oxygen environment of the placenta during early gestation, where it plays a crucial role in placental development and function [18]. However, sustained HIF1α expression after 9 weeks of gestation can lead to trophoblast cells failing to differentiate from a proliferative to an invasive phenotype, shallow invasion of trophoblasts, and insufficient myometrial spiral artery transformation, which are strongly associated with pregnancy complications [19]. On the other hand, AMPK functions as a primary regulator of energy metabolism. AMPK is activated in response to energy stress, which is characterized by increased levels of cellular AMP, ADP, or Ca^2+^ and a decline in ATP production [20]. Research has shown that AMPK plays a crucial role in maintaining the balance between trophoblast invasion and survival by controlling glucose metabolism [21]. Furthermore, elevated blood glucose levels trigger AMPK activation in the placentas of pregnant women with gestational diabetes, indicating that AMPK plays a key role in regulating glucose metabolism to maintain energy balance [22]. Finally, PPARγ is a crucial nuclear hormone receptor whose action is mediated through the heterodimerization of PPARγ with the retinoid-X-receptor (RXR) upon activation. According to previous studies, PPARγ is a crucial component for the proper progression of pregnancy, as it encompasses placental formation, fetal development, and labor [23]. Additionally, PPARγ activators, which include fatty acids and lipid metabolites, are elevated in normal pregnancies, suggesting that PPARγ may play a part in regulating maternal metabolism and immune functions during pregnancy [24].

Studies suggest that HIF1α promotes glycolysis through regulation of the glycolytic enzymes hexokinase 2 (HK2) and pyruvate kinase M2 (PKM2), as well as the glucose transporters GLUT1 and GLUT3, facilitating cellular glucose uptake [25]. Furthermore, glycolysis and glucose uptake affect the stability and activation of HIF1α in human pharyngeal carcinoma and fibrosarcoma cells and rat cardiac myocytes [25]. Conversely, AMPK activation leads to the activation of catabolic pathways that generate ATP while simultaneously inhibiting anabolic, biosynthetic pathways that consume ATP [20]. When PPARγ is activated, it initiates a series of events that ultimately lead to the regulation of glucose and lipid metabolisms. Specifically, PPARγ regulates genes associated with glucose transporters, mitochondrial biogenesis, fat storage, and fat transport [26].

The interactions between high-glucose levels and TGFβ1 have been investigated in various cell lines, and the results have demonstrated that TGFβ1 signaling affects glucose metabolism by increasing glucose uptake, controlling glycolytic enzymes, altering lactate production, and influencing oxidative phosphorylation [13,27]. However, there is no evidence regarding the effect of TGFβ1 on energy metabolism in trophoblast cells, particularly its interplay with energy-sensing regulators: HIF1α, PPARγ, and AMPK.

The aim of the present study was to investigate the potential regulatory function of TGFβ1 in counteracting metabolic disruptions caused by high-glucose conditions in trophoblast cells. By examining the expression or activation levels of SMAD2, HIF1α, PPARγ, AMPK, GLUT1, GLUT3, and specific mitochondrial respiratory chain protein subunits, as well as assessing the levels of ATP and lactate production, we aimed to explore the extent to which TGFβ1 may play a role in regulating the effects of high-glucose conditions on trophoblast cells.

## 2. Materials and Methods

### 2.1. Materials

Cell culture media and reagents such as fetal bovine serum (FBS; #090-110) and Hank’s Balanced Salt Solution (HBSS, #311-512-CL) were obtained from Wisent (St-Bruno, QC, Canada). Cell culture plates and flasks were purchased from Corning Inc., (Corning, NY, USA). TGFβ1 cytokine was purchased from Peprotech (Montreal, QC, Canada). Dimethyl sulfoxide (DMSO), bovine serum albumin (BSA), and monoclonal peroxidase-conjugated mouse anti-β-actin antibody were from Sigma Chemical Company (Oakville, ON, Canada). Protease and phosphatase inhibitors cocktail EDTA-free and the Pierce^TM^ NE-PER™ nuclear and cytoplasmic extraction reagent were purchased from Thermo Fisher Scientific (Waltham, MA, USA). VH 298, a cell-permeant inhibitor of von Hippel–Lindau disease tumor suppressor (VHL; #700410) was from Cayman Chemicals, Ann Arbor, MI, USA. Rabbit polyclonal antibodies (Abs) targeting PPARγ (#2435), HIF1α (#36169), p-SMAD2 (pS465/467; #3108), SMAD2 (#5359), p-AMPKα (pThr172; #2531), AMPK (#2532), and Lamin B1 (#13435), as well as GLUT1 (#12939), were purchased from Cell Signaling Technology (Danvers, MA, USA). The Total OXPHOS Rodent WB Antibody Cocktail (ab110413) was sourced from Abcam (Cambridge, UK), and the mouse monoclonal GLUT3 Abs (Sc-74399) from Santa Cruz Biotechnology (Cambridge, MA, USA). Rabbit polyclonal β-tubulin Abs were purchased from Abcam (#Ab6046; Waltham, MA, USA). All Abs were used at a 1:1000 dilution except for GLUT1 (1:2000) and GLUT3 (1:500) in phosphate-buffered saline (PBS) solution containing 5% BSA (PBS/5% BSA). The horseradish peroxidase-conjugated goat anti-rabbit IgG and goat anti-mouse IgG Abs (1:5000 dilution) were obtained from Bio-Rad Laboratories (Mississauga, ON, Canada). The chemiluminescence detection kit was purchased from FroggaBio (#CCH365; Concord, ON, Canada).

### 2.2. Cell Culture

The human placental choriocarcinoma JEG-3 cell line (ATCC #HTB-36; Rockville, MD, USA) was grown in RPMI-1640 cell culture media supplemented with 10% heat-inactivated FBS, 1 mM sodium pyruvate, 10 mM HEPES, and 50 μg/mL gentamicin. The cells were maintained at 37 °C in a moisture-saturated atmosphere containing 5% CO_2_. Cell culture workflow, cell passaging, and cell subculturing were performed as previously described [8,9,10]. For further experimental needs, cells were detached using trypsin, counted, and subcultured in 24- or 96-well cell culture plates. All experiments were restricted to using JEG-3 cells from passages 8 to 15 to avoid any changes in cell behavior during the study.

### 2.3. ATP Detection

The metabolic activity of JEG-3 cells was evaluated via quantitative measurement of total cellular ATP levels and achieved using an ATP detection assay kit (#700410; from Cayman Chemicals, Ann Arbor, MI, USA). Briefly, at the indicated time points, cells were lysed in ATP sample buffer and properly diluted to ensure the luminescence was within the linear range of the ATP standard curve. Cell lysates were incubated with a mixture containing D-Luciferin and Luciferase at room temperature for 20 min. The luminescence intensity was recorded at 570 nm using a microplate reader (BioTek Synergy HT; BioTek^®^ Instruments, Inc., Winooski, VT, USA). According to the manufacturer’s protocol, total ATP concentration, expressed in nM, was determined using the ATP detection standard.

### 2.4. Lactate Detection

The glycolytic activity of JEG-3 cells was determined using the Lactate-Glo Assay (#J5021, from Promega, Madison, WI, USA). In this bioluminescent assay, lactate dehydrogenase (LDH) utilizes extracellular lactate, derived from glycolysis, and NAD^+^ to produce pyruvate and NADH. In the presence of NADH, a pro-luciferin reductase substrate is converted by the reductase to luciferin, which is then used in a luciferase reaction to produce light. The light emitted in this reaction is directly proportional to the concentration of lactate in the culture medium sample. Briefly, at the indicated time points, 10 µL of cell-free culture medium samples were diluted first with 490 µL HBSS. Then, 50 µL diluted medium sample and 25 µL lactate detection reagent were mixed in 96-well plates and incubated for 60 min at room temperature. The luminescence intensity was recorded at 570 nm using the BioTek Synergy HT microplate reader. According to the technical manual, relative light unit and lactate titration curve were used to determine lactate concentration, expressed in nM.

### 2.5. Cell Viability/Proliferation

The relative cell viability/proliferation of JEG-3 cells was determined using the MTT assay as we previously described [8,9,10].

### 2.6. Subcellular Fractionation

JEG-3 cells at a cell density of 450 × 10^3^ cells/2 mL/well were seeded into 6-well plates in 10% FBS-RPMI-1640 cell culture media overnight. Cells were starved without FBS overnight, and then cultured for 24 h. After the incubation period, cytoplasmic and nuclear protein extracts were prepared according to the instructions of the Pierce^TM^ NE-PER^®^ nuclear and cytoplasmic extraction kit (#78835) and analyzed by western blot.

### 2.7. Proteins Immunodetection

JEG-3 cells were seeded for 24 h at two different cell densities, 450 × 10^3^ cells/2 mL/well into 6-well plates and 150 × 10^3^ cells/500 μL/well into 24-well plates. Cells were starved in cell culture media without FBS overnight. Cells were then used in different experimental conditions. At the indicated time points, cell protein samples were resolved using SDS-PAGE under reducing conditions, and then transferred into PVDF membranes as described [8,9,10]. Blots were probed overnight at 4 °C with rabbit polyclonal primary Abs against total (t) or phosphorylated (p) forms of SMAD2, HIF1α, PPARγ, GLUT1, and AMPK proteins, as well as a mixture of mouse polyclonal primary Abs against mitochondrial respiratory chain protein subunits and mouse monoclonal Abs against GLUT3. Membranes were then incubated with HRP-conjugated goat anti-rabbit IgG or goat anti-mouse IgG Abs at 1:5000 dilution for 1 h at room temperature. β-actin (1:40,000 dilution) and β-tubulin and Lamin B1 (1:1000 dilutions) were used as loading controls. The detected proteins were visualized as described [8,9,10]. To ensure the validity of western blot quantification, the best Ab dilution was established to avoid background or nonspecific reactions, and a serial dilution of protein samples was conducted to optimally yield positive signals falling within the linear detection range for each antibody.

### 2.8. Statistical Analysis

Data collection and statistical analysis were performed using Prism software, version 3.03 (San Diego, CA, USA), as previously described [8,9,10]. Briefly, data from at least three independent experiments were expressed as mean ± SD. A one-way ANOVA followed by Bonferroni post-tests was performed to assess the statistical correlation of data between groups. Tukey tests were performed to analyze the null hypothesis of no difference in means between two groups. A threshold of significance at *p* ≤ 0.05 was considered to indicate statistical significance.

## 3. Results

### 3.1. The Interplay Between Glucose Concentration and Metabolic Changes in Human Trophoblast Cells

**Figure 1:**
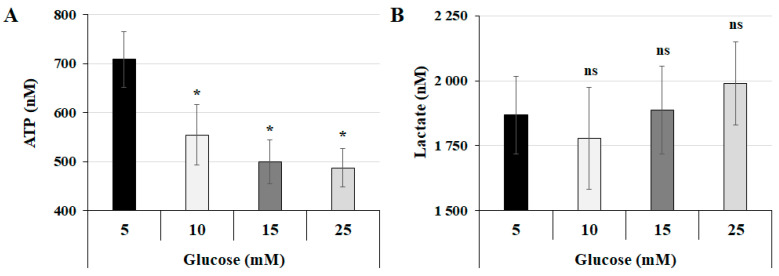
High-glucose concentration decreases total ATP production without affecting lactate production: (**A**,**B**) JEG-3 cells were cultured for 24 h in media containing normal-glucose (5 mM) or high-glucose (10, 15, and 25 mM) concentrations. (**A**) Quantitation of ATP production (*n* = 3) was assessed using the ATP luminescence detection assay kit. (**B**) Quantitation of lactate production (*n* = 3) was assessed using the Lactate-Glo^TM^ assay kit. Data are expressed as nM of ATP and lactate. * *p* < 0.05 indicates a significant difference compared to the 5 mM glucose group, and ns = nonsignificant difference.

To determine the effect of glucose concentration on metabolic regulation in the human trophoblast cell line JEG-3, cells were incubated for 24 h with increasing concentrations of glucose: 5 mM (control), 10 mM, 15 mM, and 25 mM. We assessed both ATP and lactate production, as these markers are useful for evaluating energy and carbohydrate metabolism, respectively. JEG-3 cells growing in increasing glucose concentrations exhibited a significant decrease in total ATP production immediately after the glucose concentration was doubled from 5 mM to 10 mM (Figure 1A). This decline continued gradually, reaching the lowest ATP concentration after a 24 h incubation period with 25 mM glucose. Regarding lactate production (Figure 1B), no significant changes in lactate production were observed, regardless of the glucose concentration. These findings suggest that increasing glucose concentrations does not affect the glycolytic pathway but probably induces alterations in ATP production within the mitochondria of JEG-3 cells.

### 3.2. Effect of High-Glucose Concentration on Mitochondrial Respiratory Chain Protein Expression

**Figure 2:**
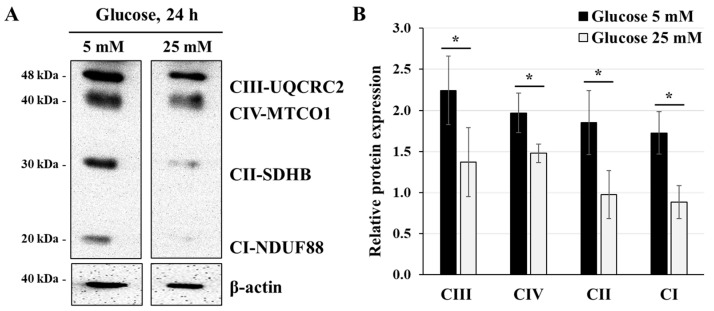
High-glucose concentration decreases the expression of mitochondrial respiratory chain proteins: (**A**,**B**) JEG-3 cells were cultured for 24 h in media containing normal-glucose (5 mM) or high-glucose (25 mM) concentrations. (**A**) Representative images of OXPHOS complexes protein subunits detection and β-actin, as assessed by western blot. (**B**) Graphical analysis showing the expression of different mitochondrial complex protein subunits relative to β-actin at 5 mM and 25 mM glucose (*n* = 3). * *p* < 0.05 denotes a significant difference between the cell groups.

Because high-glucose condition is known to reduce ATP synthesis and mitochondrial function in trophoblast cells [2,3], and that variation in the expression of mitochondrial respiratory chain proteins is linked to enhanced [28] or lowered [29] cellular ATP synthesis levels, we evaluated the expression of mitochondrial respiratory chain proteins via western blot analysis (Figure 2A). JEG-3 cells were incubated for 24 h with glucose at either 5 mM or 25 mM—the concentration that mimics conditions of hyperglycemia in vivo. The results showed that incubating JEG-3 cells with 25 mM glucose significantly reduced the expression of all mitochondrial respiratory chain protein subunits: CI-NDUF88 (2-fold), CII-SDHB (1.9-fold), CIII-UQCRC2 (1.6-fold), and CIV-MTCO1 (1.3-fold) (Figure 2B). This indicates that cells growing in high-glucose conditions lead to alterations in mitochondrial function.

### 3.3. Effect of High-Glucose Concentrations on the Expression of HIF1α and PPARγ

**Figure 3:**
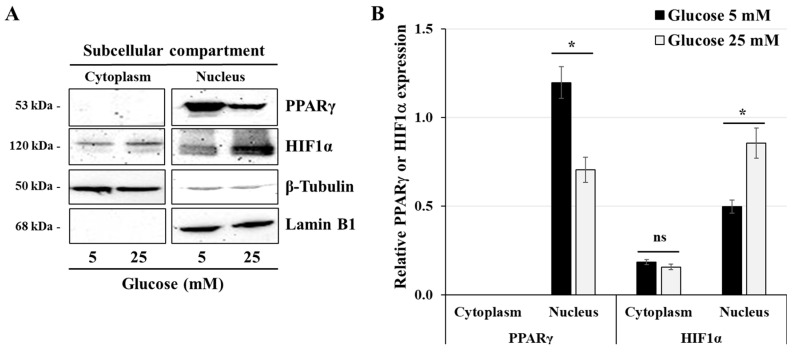
High-glucose concentration differentially regulates the nuclear expression of PPARγ and HIF1α: (**A**,**B**) JEG-3 cells were cultured for 24 h in media containing normal-glucose (5 mM) or high-glucose (25 mM) concentrations. (**A**) Representative images of PPARγ, HIF1α, β-tubulin, and Lamin B1 in the cytoplasmic and nuclear compartments of JEG-3 cells, as assessed by western blot. (**B**) Graphical analysis showing the relative expression of HIF1α and PPARγ in the cytoplasm and the nucleus at 5 mM and 25 mM glucose (*n* = 3). * *p* < 0.05 denotes a significant difference between the cell groups, and ns = nonsignificant difference.

To better understand the mechanism by which high-glucose concentration alters ATP production and mitochondrial metabolism, we investigated the expression of HIF1α and PPARγ, two key transcription factors involved in the regulation of carbohydrate metabolism. HIF1α, a transcription factor induced by hypoxia, is also known to regulate glycolytic metabolism [30]. PPARγ is a key regulator of energy metabolism, particularly in mitochondrial biogenesis [31]. In this set of experiments, proteins from cytoplasmic and nuclear compartments were extracted from JEG-3 cells that were incubated with either normal-glucose (5 mM) or high-glucose (25 mM) concentrations for 24 h. The results (Figure 3A,B) showed that growing conditions in high-glucose concentration induced a decrease in the nuclear expression of PPARγ (1.7-fold decrease) compared with the normal-glucose condition. Conversely, nuclear expression of HIF1α appears to be increased (1.7-fold increase) in JEG-3 cells after a 24 h incubation period with high-glucose concentration compared with 5 mM glucose (Figure 3A,B). Taken together, these results indicate that high-glucose concentration induces alterations in the regulation of energy metabolism in the human trophoblast JEG-3 cells. These alterations appear to be mediated through differential expression of the energy metabolic sensors HIF1α and PPARγ.

### 3.4. Effect of Glucose Concentrations on the Activation of the TGFβ1 Signaling Pathway

**Figure 4:**
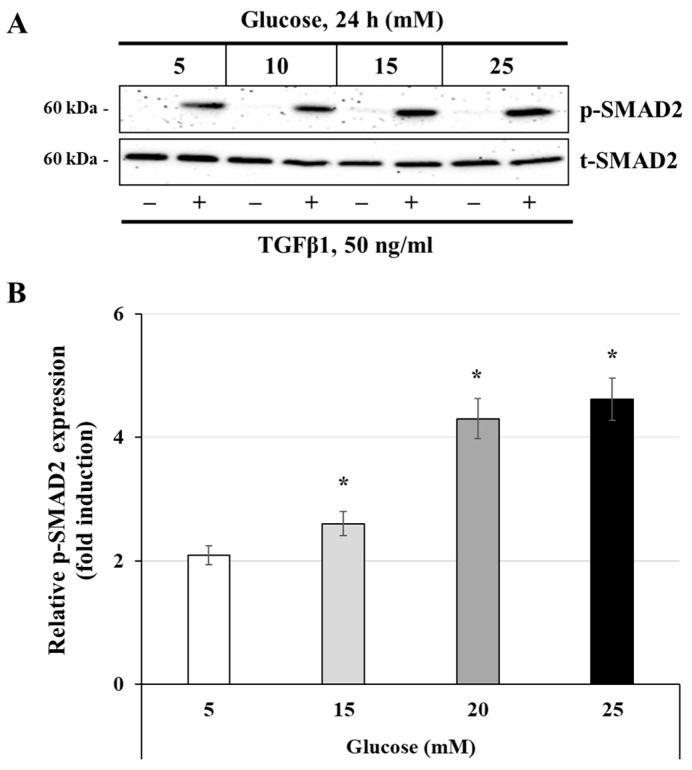
Glucose concentration regulates TGFβ1-induced SMAD2 phosphorylation: (**A**,**B**) JEG-3 cells were cultured for 24 h in media containing normal-glucose (5 mM) or high-glucose (10, 15, and 25 mM) concentrations, and then stimulated for 30 min with culture media alone (control) or with 50 ng/mL TGFβ1. (**A**) Representative images showing the expression of phosphorylated SMAD2 (p-SMAD2) and total SMAD2 (t-SMAD2), as evaluated by western blot. (**B**) Graphical analysis showing the expression levels of p-SMAD2 relative to t-SMAD2 for each glucose concentration (*n* = 3). * *p* < 0.05 denotes a significant difference compared to control.

In human trophoblast cells, TGFβ1 is recognized as a key pregnancy-related cytokine and regulates various aspects of trophoblast function such as differentiation and proliferation [32]. TGFβ1 is also known to be a main regulator of metabolism in immune cells, such as macrophages, by promoting mitochondrial metabolism over glycolytic metabolism [33]. In this set of experiments, we aimed to assess the effect of TGFβ1 on the metabolic dysregulation induced by growing conditions in high-glucose concentration. Initially, we evaluated the impact of different glucose concentrations on the phosphorylation of SMAD2, a key signal transduction molecule induced by TGFβ1. Our preliminary data indicates that SMAD2 activation was induced as early as 5 min after TGFβ1 stimulation, with a peak at 60 min and a continuing decline up to 120 min. JEG-3 cells were initially incubated for 24 h with increasing concentrations of glucose (5, 10, 15, and 25 mM). Then, cells were stimulated for 30 min with culture media alone (control) or with 50 ng/mL TGFβ1. The results shown in Figure 4A,B indicate that stimulation with TGFβ1 during 30 min resulted in significant induction of SMAD2 phosphorylation, with greater induction observed when the cells were incubated with 20 mM glucose (4.3-fold increase) and 25 mM glucose (4.6-fold increase). Overall, these results suggest that TGFβ1 signaling can be influenced in trophoblast JEG-3 cells when incubated in high-glucose conditions.

### 3.5. Effect of TGFβ1 on the Process of Glucose and Energy Metabolism

**Figure 5:**
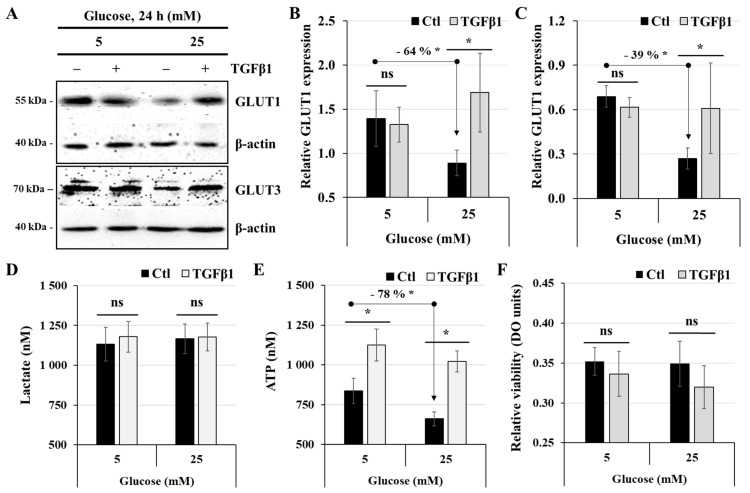
TGFβ1 differentially regulates glucose transporters expression, lactate, and ATP production: (**A–E**) JEG-3 cells were cultured for 24 h in media containing normal-glucose (5 mM) or high-glucose (25 mM) concentrations in the absence or presence of TGFβ1 at 50 ng/mL. (**A**) Representative images of GLUT1, GLUT3, and b-actin protein detection, as assessed by western blot. Graphical analysis showing the relative expression of GLUT1 (**B**) and GLUT3 (**C**) at 5 mM and 25 mM glucose. (**D**) Quantitation of lactate production was assessed using the Lactate-Glo^TM^ assay kit. (**E**) Quantitation of ATP production was assessed using the ATP luminescence detection assay kit. Data are expressed as nM of ATP and lactate (*n* = 3). (**F**) Quantitation of relative cell viability was assessed using the MTT assay (*n* = 3). Each bar represents the mean ± SD from at least two independent experiments. * *p* < 0.05 indicates a significant difference between the cell groups, and ns = nonsignificant difference.

Because the high-glucose condition significantly reduced ATP biosynthesis without affecting lactate production, we first examined the influence of glucose concentration and TGFβ1 stimulation on the expression of the glucose transporters GLUT1 and GLUT3 by western blot (Figure 5A). JEG-3 cells were thus cultured for 24 h in media containing 5 mM or 25 mM glucose in the absence (control) or the presence of TGFβ1. Our results indicate that the high-glucose condition (25 mM) decreased the expression of both glucose transporters, the relative reduction levels being evaluated at 64% for GLUT1 (Figure 5B) and 39% for GLUT3 (Figure 5C) compared to the normal-glucose condition. However, TGFβ1 stimulation significantly increased the expression of GLUT1 and GLUT3 at high-glucose concentration, at levels similar to that in the normal-glucose condition (Figure 5B,C). Next, we investigated whether TGFβ1 influences lactate and ATP production. As previously shown (Figure 1), our results confirm that JEG-3 cell incubation with normal- or high-glucose concentrations did not affect lactate production (Figure 5D) but that incubation with a high-glucose concentration significantly reduced ATP biosynthesis (Figure 5E). Moreover, we found that TGFβ1 did not affect lactate production even after a 24 h stimulation period with TGFβ1 (Figure 5D). However, as shown in Figure 5E, treatment with TGFβ1 for 24 h similarly enhanced ATP production when cells were incubated with either 5 mM glucose (1.3-fold increase) or 25 mM glucose (1.6-fold increase). To verify if the impact of TGFβ1 on cell metabolism was influenced by the effect of the hyper-glucose condition on JEG-3 cell proliferation, we evaluated the relative number of viable, metabolically active cells at 5 mM vs. 25 mM using an MTT assay. Our data indicated that neither high glucose concentrations nor the treatments with TGFꞵ1 affected JEG-3 cell proliferation. Taken together, these results indicate that TGFβ1 appears to influence energy metabolism by targeting mitochondrial function rather than lactate production in the glycolytic pathway.

### 3.6. Effect of TGFβ1 on Mitochondrial Respiratory Chain Protein Expression

**Figure 6:**
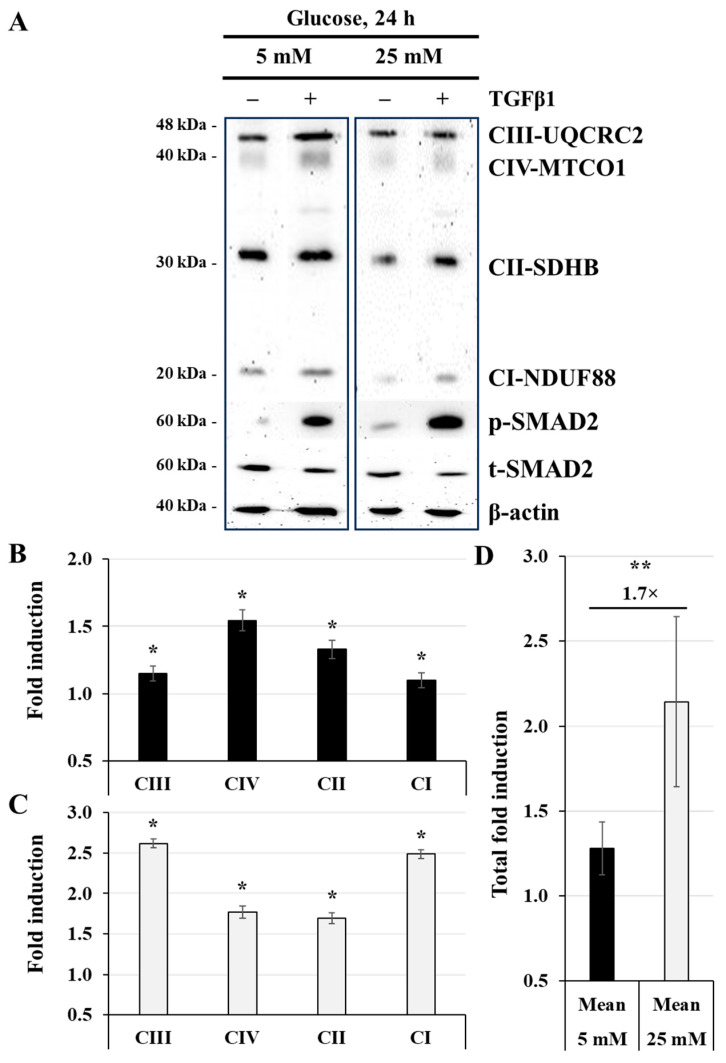
TGFβ1 enhances the expression of the mitochondrial respiratory chain proteins at normal-glucose and high-glucose concentrations: (**A**–**D**) JEG-3 cells were cultured for 24 h in media containing normal-glucose (5 mM) or high-glucose (25 mM) concentrations in the absence or presence of TGFβ1 at 50 ng/mL. (**A**) Representative images of OXPHOS complexes protein subunits detection, SMAD2 (p-SMAD2 and t-SMAD2), and β-actin, as assessed by western blot. Graphical analysis showing the expression of different mitochondrial complex protein subunits relative to β-actin at 5 mM (**B**) and 25 mM (**C**) glucose, and the summary of the total fold induction from all OXPHOS protein subunits in TGFβ1-stimulated cells relative to the control group (**D**). (**B**,**C**) * *p* < 0.05 indicates a significant difference compared to the control (*n* = 3). (**D**) ** *p* < 0.05 denotes a significant difference between 5 mM and 25 mM glucose concentrations.

To better understand the effect of TGFβ1 on the regulation of energy metabolism in JEG-3 cells, we decided to assess the expression of mitochondrial respiratory chain proteins under normal-glucose (5 mM) or high-glucose (25 mM) concentrations in the absence (control) or presence of TGFβ1 at 50 ng/mL during 24 h. The results shown in Figure 6A confirm that exposing JEG-3 cells to high glucose concentrations decreased the expression of mitochondrial respiratory chain protein subunits CI-NDUF88, CII-SDHB, CIII-UQCRC2, and CIV-MTCO1 but resulted in significant induction of p-SMAD2. Examining Figure 6B more closely, when JEG-3 cells were incubated with 5 mM glucose, treatment with TGFβ1 significantly increased the expression of OXPHOS protein subunits CI-NDUF88 (1.15-fold), CII-SDHB (1.3-fold), and CIII-UQCRC2 (1.2-fold), with a more pronounced increase in CIV-MTCO1 (1.5-fold). As shown in Figure 6C, when the cells were incubated with a high-glucose concentration (25 mM), TGFβ1 treatment enhanced the expression of OXPHOS protein subunits, with a more pronounced effect on CI-NDUF88 (2.5-fold) and CIII-UQCRC2 (2.6-fold). To better define the influence of TGFβ1 on OXPHOS protein expression at normal- and high-glucose concentrations, we evaluated the mean of total fold induction for each condition and found that TGFβ1 treatment enhanced the expression of these proteins by 1.7-fold when the cells were incubated in media containing 25 mM glucose compared to media containing 5 mM glucose (Figure 6D). Taken together, these results indicate that TGFβ1 treatment not only enhances the mitochondrial respiratory chain under control conditions but also mitigates the damage induced by high-glucose concentration.

### 3.7. Effect of TGFβ1 on the Expression of PPARγ

**Figure 7:**
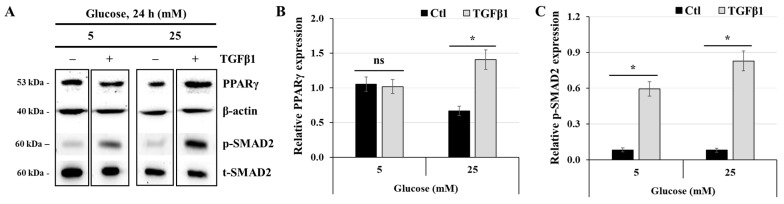
TGFβ1 increases the expression of PPARγ at high-glucose concentrations: (**A**–**C**) JEG-3 cells were cultured for 24 h in media containing normal-glucose (5 mM) or high-glucose (25 mM) concentrations in the absence or presence of TGFβ1 at 50 ng/mL. (**A**) Representative images of PPARγ, β-actin, and SMAD2 (p-SMAD2 and t-SMAD2), as assessed by western blot. Graphical analysis showing the relative expression of PPARγ (**B**) and p-SMAD2 (**C**) at 5 mM and 25 mM glucose (*n* = 3). * *p* < 0.05 indicates a significant difference between control and TGFβ1 treatments, and ns = nonsignificant difference.

To enhance our understanding of how TGFβ1 affects energy metabolism in human trophoblast cells, we determined the effect of TGFβ1 on PPARγ expression. JEG-3 cells were cultured for 24 h in media containing 5 mM or 25 mM glucose in the absence or presence of TGFβ1. Then, PPARγ expression was assessed using western blotting (Figure 7A). Our results demonstrated that cell incubation in high-glucose conditions decreased PPARγ expression (relative expression = 1.05 ± 0.08 vs. 0.67 ± 0.03; Figure 7B) but enhanced TGFβ1-induced SMAD2 phosphorylation (Figure 7C), with greater induction at 25 mM glucose (relative expression = 0.83 ± 0.09) compared with 5 mM glucose (relative expression = 0.59 ± 0.09). In normal-glucose conditions, treatment with TGFβ1 did not affect PPARγ, as reflected by similar intensity in both normal- and high-glucose concentrations (Figure 7B). However, TGFβ1 treatment significantly increased PPARγ expression under high-glucose conditions (relative expression = 1.41 ± 0.09 vs. 0.67 ± 0.09 at 5 mM). These results suggest that PPARγ may play a key role in mediating the function of TGFβ1 on energy homeostasis in JEG-3 cells under high-glucose stress conditions.

### 3.8. Effect of TGFβ1 on the Expression of HIF1α and the Activation of AMPK

**Figure 8:**
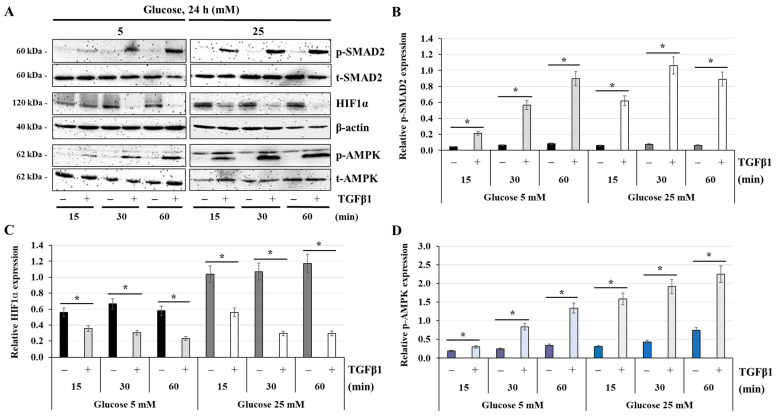
TGFβ1 decreases HIF1α expression but enhances AMPK activation at normal-glucose and high-glucose concentrations: (**A**–**D**) JEG-3 cells were cultured for 24 h in media containing normal-glucose (5 mM) or high-glucose (25 mM) concentrations, and then activated in the absence or presence of TGFβ1 at 50 ng/mL for 15, 30, or 60 min. (**A**) Representative images of SMAD2 (p-SMAD2 and t-SMAD2), HIF1α, b-actin, and AMPK (p-AMPK and t-AMPK), as assessed by western blot. Graphical analysis showing the relative expression of p-SMAD2 (**B**), HIF1α (**C**), and p-AMPK (**D**), at 5 mM and 25 mM glucose (*n* = 3). * *p* < 0.05 indicates a significant difference between the cell groups.

To further elucidate the mechanisms of TGFβ1 in the maintenance of ATP balance under normal-glucose and high-glucose stress conditions, we investigated the regulatory role of TGFβ1 on HIF1α expression and AMPK activation. In this set of experiments, JEG-3 cells were cultured for 24 h in normal-glucose (5 mM) or high-glucose conditions (25 mM), and then activated in the absence (control) or presence of TGFβ1 at 50 ng/mL for 15, 30, or 60 min (Figure 8A). As expected, treatment with TGFβ1 induced p-SMAD2 with greater phosphorylation levels at 25 mM glucose (relative expression, control = 0.066 ± 0.007 vs. TGFβ1 = 0.57 ± 0.08 at t = 30 min; Figure 8B) compared with 5 mM glucose (relative expression, control = 0.075 ± 0.008 vs. TGFβ1 = 1.062 ± 0.074 at t = 30 min; Figure 8B). Notably, JEG-3 cells basally expressed higher levels of HIF1α protein in both normal- and high-glucose conditions (Figure 8A,C). However, basal levels of HIF1α expression increased in high-glucose condition (relative expression, 1.07 ± 0.11 vs. 0.31± 0.02 in 5 mM glucose at t = 30 min; Figure 8C), while AMPK activation was unaffected in both normal- and high-glucose conditions (Figure 8D). In contrast, TGFβ1 treatment induced HIF1α degradation, an effect observed beginning at t = 15 min and continuing until the end of the experiment; this effect appears to be dependent on glucose concentration in the media, with greater degradation levels with 25 mM glucose (relative expression, control = 1.17 ± 0.11 vs. TGFβ1 = 0.29 ± 0.04 at t = 60 min; Figure 8C) compared with 5 mM glucose (relative expression, control = 0.58 ± 0.06 vs. TGFβ1 = 0.23 ± 0.02 at t = 60 min; Figure 8C). The degradation of HIF1α was mirrored by the induction of p-AMPK via TGFβ1, and again, with greater phosphorylation levels at 25 mM glucose (relative expression, control = 0.74 ± 0.05 vs. TGFβ1 = 2.25 ± 0.13 at t = 60 min; Figure 8D) compared with 5 mM glucose (relative expression, control = 0.34 ± 0.03 vs. TGFβ1 = 1.34 ± 0.13 at t = 60 min; Figure 8D). Taken together, these results indicate that TGFβ1 restores the energy homeostasis of JEG-3 cells under high-glucose stress conditions by inducing AMPK activation and HIF1α degradation.

### 3.9. The Von Hippel–Lindau Protein Antagonist VH298 Blocks TGFβ1-Induced HIF1α Degradation

**Figure 9:**
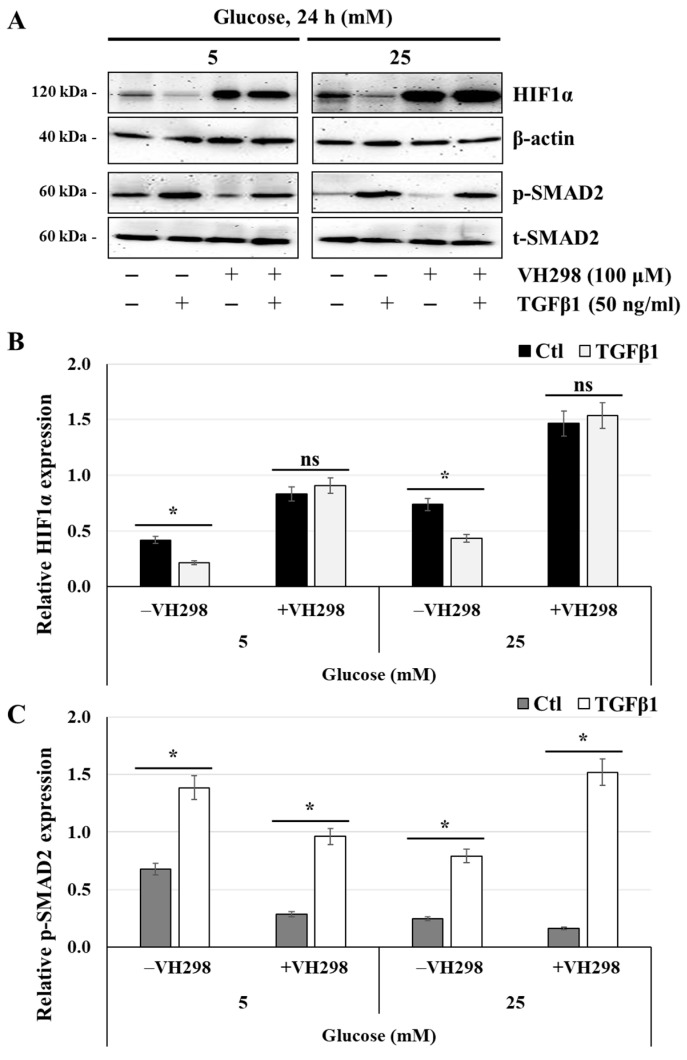
TGFβ1-induced HIF1α degradation is blocked in the presence of VH298, a specific inhibitor of ubiquitylation and proteasomal degradation of HIF1α: (**A**–**C**) JEG-3 cells were cultured for 24 h in media containing normal-glucose (5 mM) or high-glucose (25 mM) concentrations and then activated for 60 min with TGFβ1 in the absence or presence of VH298. (**A**) Representative images of HIF1α, β-actin, and SMAD2 (p-SMAD2 and t-SMAD2), as assessed by western blot. Graphical analysis showing the relative expression of HIF1α (**B**) and p-SMAD2 (**C**) at 5 mM and 25 mM glucose (*n* = 3). * *p* < 0.05 indicates a significant difference between the cell groups, and ns = nonsignificant difference.

Under normoxia, HIF1α is maintained at very low levels via proline-hydroxylation by PHD proteins, allowing their recognition and polyubiquitination by the Von Hippel–Lindau (VHL) Cullin RING E3 ubiquitin ligase complex and subsequent proteasomal degradation [17]. To elucidate the mechanisms by which TGFβ1 induces HIF1α degradation, JEG-3 cells were cultured for 24 h in media containing glucose at 5 mM or 25 mM and then activated for 60 min with TGFβ1 in the absence or presence of VH298, a potent and specific VHL inhibitor that stabilizes HIF1α by blockade of the VHL:HIF protein–protein interaction downstream of HIF1α hydroxylation by PHD enzymes. As established above (Figure 8), the basal expression levels of HIF1α significantly decreased in both normal- and high-glucose conditions after TGFβ1 stimulation (Figure 9A,B), an effect that was mirrored by enhanced levels of TGFβ1-induced SMAD2 phosphorylation (Figure 8A,C). However, treatment with VH298 molecules induced HIF1α stabilization, with greater expression levels at 25 mM glucose compared with 5 mM glucose (Figure 9A,B). Importantly, the protein expression levels of HIF1α remained stable and unchanged even in the presence of TGFβ1 (Figure 9B). Moreover, the presence of VH298 did not affect the levels of TGFβ1-induced SMAD2 phosphorylation, which were significantly increased in both normal- and high-glucose conditions after TGFβ1 stimulation (Figure 9C). These results suggest that HIF1α protein stabilisation is not caused by a blockade of SMAD2 phosphorylation by VH298 molecules and that TGFβ1 might induce HIF1α proteasomal degradation via enhanced VHL or PHD enzyme activities.

## 4. Discussion

The process of placental transport and metabolism of nutrients essential for fetal development is metabolically expensive and requires abundant oxygen consumption and ATP production, primarily synthesized through glycolysis and oxidative phosphorylation [34]. Glucose is principally the energy source for the placenta and fetus, and all the glucose supplied to the placenta-fetal unit originates from the maternal glucose pool, which is produced by maternal gluconeogenesis or ingested through the pregnant woman’s diet [35]. However, elevated glucose levels at the fetal–maternal interface have been linked to poor maternal and perinatal outcomes. In fact, hyperglycemia is the most common medical condition affecting pregnancy, and its occurrence is rising globally in tandem with the dual epidemics of obesity and diabetes [36]. The potential outcomes of hyperglycemia in pregnancies with diabetes mellitus (DM) and gestational diabetes mellitus (GDM) include preterm birth, preeclampsia, and stillbirth. However, fetal overgrowth and macrosomia are the most common adverse effects [37]. Lipid metabolism is also altered in placentas from DM and GDM women. Thus, it is proposed that maternal hyperglycemia is a contributing factor to fetal macrosomia by enhancing substrate availability to the fetus and stimulating adipose tissue formation and excessive growth [38].

Hyperglycemia can impair spiral artery remodeling, increasing the risk of pregnancy complications, such as miscarriage, cardiac and renal malformations, and rare neural conditions, such as sacral agenesis [2]. It negatively affects EVT cell function in uterine spiral artery remodeling by disrupting trophoblast proliferation, migration, invasion, hormone and angiogenic factor release, and communication between trophoblasts and immune cells [2].

In this study, we showed that high-glucose concentrations induce alterations in the regulation of energy metabolism in human trophoblast JEG-3 cells, a model of placental EVT cells. Notably, these in vitro hyperglycemic conditions reduce ATP synthesis and mitochondrial function without affecting lactate production via the glycolytic pathway. Previous research on trophoblast cells from normal and GDM placentas has shown that a high glucose concentration (25 mM) significantly reduces the glycolytic capacity and ATP production of cytotrophoblasts, particularly EVT cells [3]. EVT cells are noted for higher oxygen consumption and glycolysis compared to fusogenic VST cells. Additionally, the mitochondrial respiratory capacity of VST cells from both normal and GDM placentas remains unaffected by glucose level variations. However, VST cells from GDM placentas exhibit significantly lower levels of β-hCG and other syncytialization markers, including GCM1 and syncytin-1, compared to those from normal placentas [3]. According to the results of the microarray transcriptome analysis, DM is associated with alterations in gene expression at critical stages of placental energy metabolism, with 67% of the changes affecting lipid pathways and 9% affecting glucose pathways [39]. Furthermore, pregnant women with GDM show preferential activation of lipid genes [39]. Human trophoblast BeWo cells, a model of placental VST cells, were cultured in 5 or 25 mM glucose to identify the functional biochemical pathways perturbed by high glucose levels [40]. Among the pathway networks affected by high-glucose conditions, this study highlighted the phosphoinositide 3-kinase (PI3K) cascade, glucose metabolism, peroxisomal lipid metabolism, phospholipid metabolism, bone morphogenetic protein (BMP)/SMAD2 cascade, regulation of lipid metabolism by peroxisome proliferator-activated receptors (PPARs), and cellular response to stress [40].

Although the precise mechanism by which high glucose concentrations in EVT suppress metabolic activity and mitochondrial oxidative phosphorylation remains largely unknown, our results suggest that these changes may be mediated by reduced expression levels of mitochondrial respiratory chain proteins and PPARγ, coinciding with increased HIF1α expression.

Given the critical role that TGFβ1 plays in multiple aspects of cell metabolism [13], we hypothesized that this cytokine regulates energy metabolism in trophoblast cells by influencing the expression of key energy metabolic sensors, such as HIF1α, PPARγ, and AMPK, under high-glucose conditions. Our results showed that TGFβ1 stimulation led to increased levels of SMAD2 phosphorylation when the cells were incubated in high-glucose conditions (15 mM and 25 mM). This outcome aligns with prior research demonstrating that prolonged exposure to high glucose levels triggers a range of physiological and pathophysiological changes, activating various signaling pathways that often disrupt the function of cells, tissues, and organ systems [41]. Hyperglycemia can activate protein kinase C (PKC) in malignant cells, leading to the activation of various pathways including Akt, TGFβ/SMADs, and NFκB. These pathways work together to control cancer cell metabolism and behavior, such as proliferation, migration, invasion, and recurrence [42]. As research suggests, elevated glucose levels have been found to boost the cell membrane concentrations of TβRI and TβRII and to promote the activation of latent TGFβ by matrix metalloproteinases [13]. This, in turn, triggers the Akt-mTOR pathway and leads to enlargement of fibroblasts and epithelial cells. Furthermore, TGFβ signaling plays a role in regulating other components of the glycolytic pathway [13].

Our findings indicated that TGFβ1 restores the energy homeostasis of JEG-3 cells under high-glucose stress conditions. Specifically, TGFβ1 treatment enhances ATP production and mitochondrial respiratory chain protein expression under high-glucose concentrations. While high-glucose stress condition does not affect lactate production, even after TGFβ1 stimulation, we found that TGFβ1 restores the expression levels of GLUT1 and GLUT3 to levels similar to those observed in normal-glucose conditions. These results are consistent with previous research showing that TGFβ1 improves the activity/expression of GLUT1 and HK2 in different cell types, thus favoring glucose capture and energy production [13]. Moreover, it was established that TGFβ1 is responsible for significant metabolic reprogramming in normal and cancerous cells by promoting mitochondrial ATP production [14] and increasing mitochondrial activity, as evidenced by the higher mitochondrial DNA copy number and ROS levels [43]. Furthermore, TGFβ1 activates the AMPK pathway, which promotes fatty acid oxidation and inhibits anabolic pathways [44]. It is proposed that this effect on mitochondrial metabolism can help normal and cancer cells maintain their energy requirements and metabolic adaptations under metabolic stress conditions.

The impact of TGFβ1 on trophoblast metabolism is not extensively investigated. Here, we demonstrated that TGFβ1 treatment of trophoblast JEG-3 cells resulted in the degradation of HIF1α under normal- and high-glucose concentrations. Although prior studies have demonstrated that TGFβ1 induces HIF1α stability under hypoxic and normoxic conditions by inhibiting the prolyl hydroxylase domain protein PHD2, an oxygen sensor that typically promotes HIF1α degradation [45], notably, the stability and function of HIF1α are also regulated by hyperglycemia through interference with the degradation of HIF1α triggered by PHD enzymes [46]. The findings suggest the presence of dual effects of TGFβ1 on HIF1α stability and degradation in different physiological and pathological contexts. In this study, we demonstrated that TGFβ1-induced HIF1α degradation is blocked in the presence of VH298, a potent and specific VHL inhibitor that disrupts VHL:HIF protein–protein interaction and subsequent HIF1α proteasomal degradation, suggesting that TGFβ1 might induce HIF1α proteasomal degradation via enhanced VHL or PHD enzyme activities. Nonetheless, additional research is necessary to understand the precise mechanism by which TGFβ1 causes HIF1α degradation in JEG-3 cells.

The impact of TGFβ1 on HIF1α in JEG-3 cells was accompanied by the activation of AMPK, regardless of glucose concentration. This outcome aligns with research suggesting that TGFβ1 can indirectly activate AMPK through various signaling pathways [47]. Particularly, IGF-I and IGFBP-2 have been shown to stimulate AMPK activation while assisting in its modulation. AMPK activity plays a vital role in maintaining energy balance and metabolic regulation [47].

Conversely, our findings demonstrated that glucose concentration affected the effect of TGFβ1 on PPARγ. Specifically, we found that TGFβ1 did not affect PPARγ expression under normal glucose levels. These results indicate a potential interaction between PPARγ and AMPK in relation to the effect of TGFβ1 on ATP production. AMPK activation enhances the expression of genes related to fatty acid oxidation (FAO) through interaction with PPARγ, thereby improving mitochondrial function and ATP production. Independent research indicates that combined activation of AMPK and PPARγ synergistically upregulates FAO genes, boosting ATP levels in macrophages [48,49]. The AMPK-PPARγ coactivator 1α (PGC-1α) pathway is crucial for mitochondrial biogenesis and function, as it enhances PPARγ activity, thereby increasing the expression of mitochondrial genes and promoting ATP production. This pathway is vital for maintaining energy homeostasis in macrophages under metabolic stress [48,49].

Our results indicate that TGFβ1 restores PPARγ expression under high glucose concentrations, suggesting that PPARγ is involved in enhancing ATP production in JEG-3 cells under high glucose stress. Although earlier investigations have revealed that the activation of PPARγ by agonists in β-cells can promote mitochondrial energy metabolism and improve ATP production, this regulation helps manage glucose-stimulated insulin secretion under lipotoxic conditions [50,51]. Investigations have revealed that activation of PPARγ in prostate cancer cells increased mitochondrial biogenesis and ATP levels by upregulating AKT3, which enhanced the nuclear localization of PGC1α [50,51].

## 5. Conclusions

**Figure 10:**
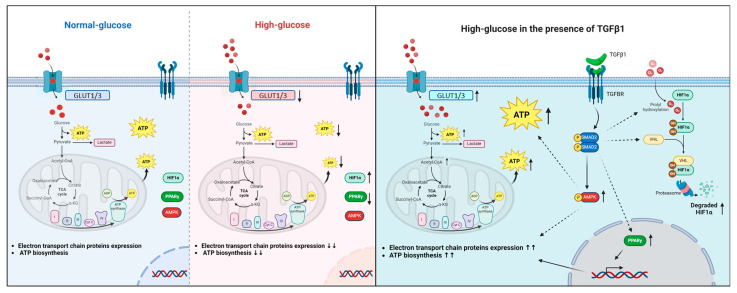
Graphical representation suggesting a protective effect of TGFβ1/SMAD signaling pathways in trophoblast cells under high-glucose stress conditions by restoring ATP production via the induction of HIF1α degradation, AMPK activation, and restoring PPARγ, GLUT1/3, and electron transport chain protein expression. ↑ means increase and ↓ means decrease.

Elevated blood glucose levels at the fetal–maternal interface are associated with problems in placental trophoblasts and an increased risk of pregnancy-related complications. Our study suggests that TGFβ1 may play a protective role against the negative effects of high-glucose concentrations on trophoblast JEG-3 cells. Our results showed that high-glucose treatments disrupted energy and mitochondrial metabolism, which was indicated by a decrease in ATP production without affecting lactate production. At the molecular level, this was demonstrated by an increase in HIF1α expression and nuclear localization, a decrease in PPARγ expression and nuclear localization, and a decrease in the expression of GLUT1, GLUT3, and respiratory chain proteins. Our study is the first to demonstrate that TGFβ1 can have a protective effect by restoring ATP production under high-glucose stress conditions by inducing HIF1α degradation, AMPK activation, and restoring PPARγ and respiratory chain protein expression (Figure 10).

These results are significant because they add to the list of TGFβ1’s effects during pregnancy. It is important to note that TGFβ plays a pleiotropic role during pregnancy with both beneficial and pathological effects [15]. On the one hand, TGFβ1 is essential for a successful pregnancy by regulating immune tolerance, trophoblast invasion, and placental development. Clinical studies also emphasize its crucial role in maintaining pregnancy health. For example, lower secretion levels of TGFβ were found in the serum of recurrent pregnancy loss patients compared to healthy pregnant women [52]. In unexplained recurrent spontaneous abortion pregnancies, the level of TGFβ in the decidual and villous tissues was significantly lower than the samples from normal pregnancies [53]. On the other hand, elevated TGFβ levels have been correlated with severe preeclampsia and adverse maternal and fetal outcomes [15]. TGFβ1 was also significantly increased in the serum of GDM patients [54]. In this context, recent studies have identified shared pathways between diabetes and preeclampsia, including TGFβ involvement in the PI3K/Akt signaling pathway, which contributes to endothelial dysfunction and placental abnormalities [55]. In contrast, it is reported that high TGFβ3 levels, but not TGFβ1 or TGFβ2 levels, can suppress trophoblast outgrowth, leading to shallow placental invasion and an increased risk of preeclampsia [56]. Subsequently, further research is needed to better understand the role of different TGFβ isoforms in regulating glucose metabolism and energy balance in the placenta and in trophoblast cells and its potential use in protection against gestational complications.

## 6. Limitations of the Study

The trophoblast JEG-3 cell is a choriocarcinoma cell line, which presents a limitation in the study design that impacts the ability to interpret and generalize from our research results. In fact, due to ethical restrictions and the limited availability of human tissues, trophoblast cell lines are generally used to investigate many aspects of trophoblast biology and metabolism. According to a study by Lee et al. (2016), JEG-3 cells share some expression characteristics with primary human trophoblast cells, particularly the expression of C19MC miRNAs [57]. Additionally, KRT7, GATA3, and TFAP2C are emphasized as reliable markers for identifying primary trophoblasts, supporting the characterization of JEG-3 cells. Additionally, a study by Weiss et al. (2001) showed that compared to other immortalized cell lines, JEG-3 cells exhibit higher expression of glucose transporters GLUT1 and GLUT3, suggesting a greater capacity for glucose uptake [58].

Future in vitro investigations using human trophoblast stem cells or placental explants will further provide insights into fundamental biological effects and the regulatory role of TGFβ1 in different metabolic conditions. We also plan to confirm whether these TGFβ1 mechanisms operate in an in vivo model. Moreover, the complex and sometimes contradictory effects of TGFβ on pregnancy also underscore the need to confirm our results under more physiologically relevant experimental conditions, particularly by better mimicking the maternal–fetal interface.

## Data Availability

The original contributions presented in this study are included in the article. Further inquiries can be directed to the corresponding author(s).
